# Research Advances in the Impact of Probiotic Supplementation on Ulcerative Colitis Management

**DOI:** 10.3390/nu17243838

**Published:** 2025-12-08

**Authors:** Yangyang Xu, Jie Zhang, Ruitao Cai, Chuyang Wei, Yuwei Chen, Xiaoyong Liu

**Affiliations:** 1 School of Biological Science and Technology, University of Jinan, Jinan 250024, China; 17829359413@163.com (Y.X.); jie_zhang1229@163.com (J.Z.); crt17853319565@163.com (R.C.); wei_chaya@163.com (C.W.); 2 Qilu Zhongke Institute of Space Science and Utilization, Jinan 250013, China

**Keywords:** ulcerative colitis (UC), probiotics, intestinal mucosa, intestinal microbiota

## Abstract

Ulcerative colitis (UC) is a chronic, non-specific inflammatory bowel disease with an unknown etiology. The primary symptoms include abdominal pain, diarrhea, and mucopurulent bloody stools, which manifest in recurrent episodes and often resist therapeutic interventions. Recent research has increasingly emphasized the potential role of probiotics in the management of UC, revealing a significant dysbiosis in the intestinal microbiota of affected individuals. Probiotic supplementation has demonstrated efficacy in alleviating UC symptoms through various mechanisms. Probiotics contribute to the restoration of the intestinal microecosystem balance by promoting beneficial bacteria and inhibiting pathogenic strains. They also play a role in modulating the immune response, thereby reducing inflammation by suppressing pro-inflammatory cytokines and enhancing anti-inflammatory cytokines, which collectively help mitigate intestinal inflammation. Furthermore, certain probiotics have been shown to improve intestinal barrier function, preventing the invasion of pathogenic microorganisms and enhancing intestinal permeability. Although numerous animal studies and clinical trials have validated the positive effects of probiotics on UC, the degree of efficacy varies among different strains. This article reviews the mechanisms and clinical applications of probiotics in the management of UC, offering new insights for its clinical treatment.

## 1. Introduction

Ulcerative colitis (UC) is a chronic, non-specific inflammatory disease of the intestines that has seen a significant rise in global incidence in recent years [1]. Epidemiological data indicate that in developed countries, including those in Europe and the United States, the incidence of UC ranges from 10 to 20 cases per 100,000 individuals, with a prevalence rate of approximately 80 to 160 cases per 100,000 individuals. Although China is classified as a low-incidence area for UC, the westernization of lifestyle and changes in environmental factors have contributed to a gradual increase in both incidence and prevalence, significantly impacting the quality of life and both the physical and mental health of patients [2]. UC primarily affects the colonic and rectal mucosa and submucosa, leading to a diverse array of clinical symptoms. Common symptoms include persistent or recurrent diarrhea, mucopurulent bloody stool, abdominal pain, and tenesmus [3]. These symptoms not only cause discomfort but also significantly disrupt patients’ daily lives, work, and social interactions. Furthermore, long-term intestinal inflammation may lead to severe complications such as toxic megacolon, intestinal bleeding, intestinal perforation, and colorectal cancer, posing a substantial threat to patients’ health and well-being [4].

Currently, the clinical management of UC primarily involves the use of aminosalicylates, glucocorticoids, immunosuppressants, and biologics. While these agents can induce remission, each has its limitations. Aminosalicylates are effective for mild to moderate UC but are often inadequate for severe cases [5]. Glucocorticoids provide rapid anti-inflammatory effects but carry risks of adverse events with long-term use, including osteoporosis, hyperglycemia, and increased susceptibility to infections [6]. Immunosuppressants may take weeks to months to become effective and can lead to hepatorenal toxicity or bone marrow suppression [7]. Biologics, while targeted, are often costly, and some patients experience secondary failure or hypersensitivity reactions [8,9,10]. Additionally, these treatments generally focus on symptom control rather than offering a cure, with many patients experiencing relapses that necessitate long-term therapy. This scenario underscores the need for complementary strategies that address underlying factors such as dysbiosis of the gut microbiota.

With advancements in research regarding the relationship between intestinal microecology and human health, probiotics have emerged as a promising supportive option, attracting increasing attention. Probiotics are live microorganisms that confer beneficial effects on the host’s health when consumed in adequate amounts. Their mechanisms of action primarily involve regulating the balance of intestinal microbiota, enhancing intestinal mucosal barrier function, inhibiting inflammatory responses, and modulating immune function [11]. Intestinal microbiota are believed to play a pivotal role in the pathogenesis of colitis [12]. Consequently, probiotic intervention is regarded as a safe and potentially effective adjunct to conventional treatment for UC [13]. Research indicates that probiotics improve intestinal barrier function by balancing or regulating intestinal microbiota and influencing the host’s innate and adaptive immune responses, thereby promoting intestinal health during its transit through the gastrointestinal tract [14]. Clinical studies on probiotic intervention in UC patients have demonstrated promising results, including prolonged remission rates, reduced gastrointestinal discomfort, and enhanced subjective well-being [15]. However, despite these encouraging findings, the current evidence base remains insufficient to support a definitive judgment on efficacy due to variability and inconsistency in most clinical studies regarding the role of probiotics [16]. As highlighted in existing literature, this uncertainty surrounding their alleviating efficacy poses a substantial challenge to the innovation and application of probiotics [17].

A thorough investigation into the effects of probiotics on UC is both scientifically significant and clinically valuable. This research can enhance our understanding of the pathogenesis of UC, clarify the crucial role of intestinal microecology in its onset and progression, and provide a theoretical foundation for developing more effective management strategies. Additionally, examining the mechanisms of action and clinical efficacy of probiotics may help identify specific strains or combinations that offer the most supportive benefits. This could lead to safer and more effective supportive options for UC patients, ultimately improving their quality of life and reducing both social and medical burdens.

## 2. The Pathogenesis of UC

**Figure 1:**
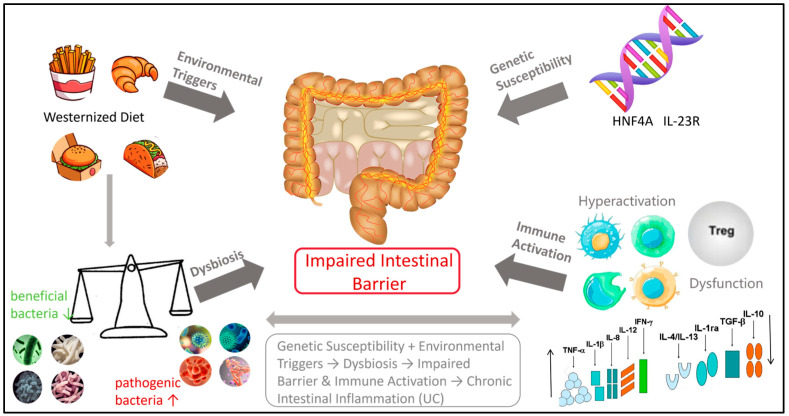
Influencing factors of UC.

As illustrated in Figure 1, the pathogenesis of UC is influenced by a range of factors, including genetic predispositions, environmental influences, immune responses, and imbalances in intestinal microbiota [18,19,20]. These factors are interrelated and interact with one another, contributing to the onset and progression of intestinal inflammation [21].

Genetic factors play a role in the pathogenesis of UC, though their influence is relatively modest. Genome-wide association studies (GWAS) have identified several risk gene loci, including HNF4A [22] and IL-23R [23]. These genes primarily contribute to immune regulation and mucosal barrier function; however, genetic factors account for only approximately 7–10% of disease susceptibility [22,24].

Environmental factors, particularly dietary influences, are significant external contributors to the disease. A Westernized diet characterized by high sugar, high fat, and additives (such as emulsifiers and artificial sweeteners) can substantially alter the composition of intestinal flora. This dietary shift leads to an increase in pro-inflammatory bacteria (such as *Proteobacteria*), a reduction in beneficial bacteria (such as *Bifidobacterium* and *Lactobacillus*), and a decline in the production of short-chain fatty acids (SCFAs) [25,26]. This dysbiosis of intestinal flora is a critical factor in the pathogenesis of UC.

A healthy intestinal microbiome provides energy for intestinal epithelial cells, maintains barrier integrity, and inhibits pro-inflammatory pathways, such as NF-κB, through the production of metabolites, including SCFAs (such as butyric acid) and vitamins. This process is essential for establishing an environment conducive to immune tolerance [27,28]. Patients with UC exhibit decreased bacterial diversity and reduced SCFA levels, which compromise intestinal barrier function and increase intestinal permeability [27,29,30,31].

Ultimately, the abnormal activation of the immune system is a direct cause of tissue damage. In the context of genetic susceptibility and dysbiosis, the intestinal mucosal immune system’s response to intracavitary antigens becomes dysregulated. This is characterized by the excessive activation of pro-inflammatory T cell subsets (such as Th1 and Th17) and their associated cytokines (such as IFN-γ and IL-17), while the regulatory T cells (Treg) exhibit impaired function and fail to effectively inhibit inflammation [32,33]. Concurrently, innate immune cells (such as macrophages and neutrophils) are recruited in large numbers and release inflammatory mediators, including reactive oxygen species and proteases, resulting in intestinal epithelial damage, erosion, and ulceration, which contribute to chronic inflammation [32].

In summary, the incidence of UC is influenced by an individual’s genetic background. It is triggered by adverse environmental factors, particularly dietary influences, and results from a chain reaction involving intestinal flora imbalance, mucosal barrier damage, and abnormal immune activation.

## 3. The Relieving Effect of Probiotics on UC

### 3.1. Concept, Classification and Physiological Function of Probiotics

Probiotics are active microbial communities that confer health benefits to the host when consumed in adequate amounts. These microorganisms primarily colonize the human intestine and reproductive system, playing various physiological roles by regulating the host’s microecological balance [34]. According to the World Health Organization (WHO) and the Food and Agriculture Organization of the United Nations (FAO), probiotics must meet three essential criteria: they must be active and capable of surviving and reproducing within the host; a sufficient intake must be achieved to elicit health effects; and their health-promoting effects must be scientifically validated.

These beneficial microorganisms are crucial for maintaining digestive health, improving metabolic function, and enhancing the body’s defenses. They achieve this by competitively inhibiting harmful bacteria, strengthening intestinal barrier function, and modulating immune responses, among other mechanisms. Recent studies indicate that different strains of probiotics may offer specific health benefits, underscoring the importance of selecting appropriate strains and dosages tailored to individual needs [34].

In microbiological classification, probiotics primarily include *Lactobacillus*, *Lacticaseibacillus*, *Bifidobacterium*, *Bacillus*, and *Saccharomyces*, among others. As illustrated in Table 1, each category encompasses various strains that exhibit distinct morphological, physiological, and functional differences. Emerging evidence also suggests that certain probiotics, often referred to as “psychobiotics”, may improve mood and alleviate anxiety in patients with ulcerative colitis via the gut–brain axis, although clinical data remain limited.

Common species of *Lactobacillus* include *Lactobacillus acidophilus* and others [28]. *Lactobacillus acidophilus* is recognized for its strong acid resistance and ability to colonize the human intestine and oral cavity, enabling it to compete with pathogens for resources [35]. Additionally, it exhibits bactericidal effects, making it valuable in addressing gastric diseases caused by *Helicobacter pylori* infection. By secreting lactic acid, it lowers intestinal pH and inhibits harmful bacteria [36].

Common species of *Lacticaseibacillus* include *Lacticaseibacillus rhamnosus* and *Lacticaseibacillus casei*. *Lacticaseibacillus rhamnosus* demonstrates the ability to tolerate bile and gastric acid, maintaining stability within the intestine. It adheres to intestinal epithelial cells, regulates the expression of tight junction proteins, enhances the biological barrier against pathogenic bacteria, and improves gut microbiota balance [37]. Furthermore, when combined with other probiotics, it aids in relieving constipation and other intestinal discomforts [38].

*Lacticaseibacillus casei* has applications in improving arthritis by activating intestinal immune cells, stimulating the production of IL-10 and IL-4, and inhibiting TNF-α, IL-1β, and IL-6, thereby balancing anti-inflammatory and pro-inflammatory factors [39]. Animal studies indicate that it can reduce joint swelling and pain while enhancing joint function [40]. Common species of *Bifidobacterium* include *Bifidobacterium infantis* and *Bifidobacterium longum* [41]. *Bifidobacterium infantis* regulates intestinal microbiota and barrier function, reduces endotoxin levels in the blood, mitigates liver inflammation, and enhances liver protection [42]. When combined with Lactobacillus, it has shown effectiveness in supporting the management of alcohol-induced liver injury [43]. *Bifidobacterium longum* adapts well to the intestinal environment and works synergistically with other probiotics. It promotes the proliferation of intestinal mucosal cells, enhances intestinal barrier function, and prevents the invasion of toxins and harmful bacteria [44]. Additionally, it helps regulate the secretion of intestinal neurotransmitters, improves intestinal dysfunction, and may alleviate issues related to emotional stress [49]. Mental probiotics are defined as living microorganisms that can positively impact the host’s mental health following ingestion [50]. They serve a regulatory function through the gut–brain axis, a bidirectional communication network connecting the intestine and the brain. In patients with UC, chronic intestinal inflammation and dysbiosis can influence the central nervous system’s function via the gut–brain axis, resulting in a heightened incidence of emotional disorders such as anxiety and depression [51]. Certain psychiatric probiotics, including specific strains of *Bifidobacterium longum* and *Lacticaseibacillus rhamnosus*, have demonstrated the ability to regulate the synthesis and metabolism of intestinal neurotransmitters such as 5-hydroxytryptamine and γ-aminobutyric acid (GABA), thereby improving emotional states and stress responses [50,52]. This offers a novel perspective on the comprehensive management of both physical and mental symptoms in UC patients at the microecological level.

*Bacillus* predominantly comprises *Bacillus subtilis*, which can form spores and endure harsh environments. Once in the intestine, it rapidly consumes free oxygen, thereby creating an anaerobic environment that promotes the growth of beneficial anaerobic bacteria [45]. Additionally, it secretes enzymes such as protease and amylase to facilitate food breakdown, enhance nutritional utilization, and alleviate abdominal discomfort associated with poor digestion [46]. *Saccharomyces* includes *Saccharomyces boulardii*, which exhibits antimicrobial and antitoxin properties while providing nutritional benefits to the intestinal mucosa. It enhances microecological balance, mitigates digestive dysfunctions such as diarrhea and constipation, and regulates intestinal motility by modulating the microbiota [47,48].

### 3.2. The Mechanism of Probiotics Alleviating UC

**Figure 2:**
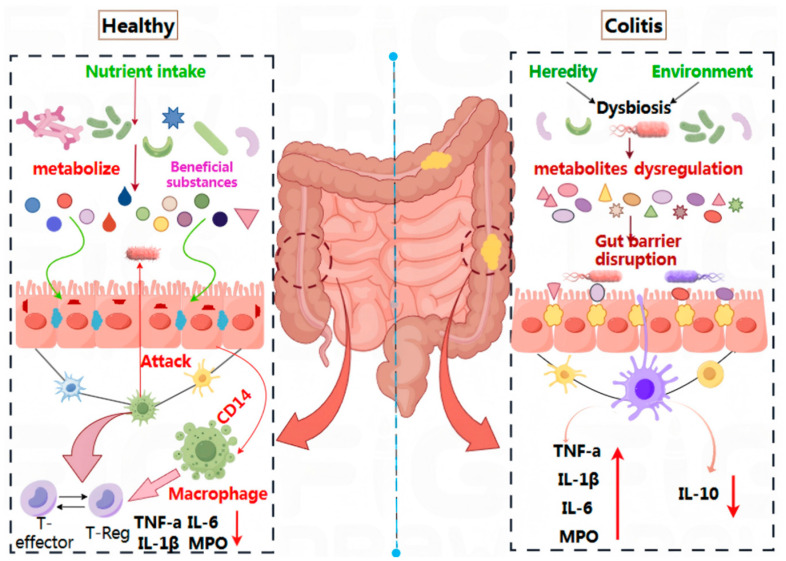
The mechanism of probiotics on the remission of UC.

Recent years have witnessed significant advancements in our understanding of the relationship between intestinal microecology and human health [53]. Probiotics, as active microorganisms, have emerged as an innovative supportive approach and are increasingly becoming a focal point of research within the medical field. Their therapeutic effects on UC have been validated through both animal studies and clinical trials. Figure 2 illustrates that in a healthy intestinal environment, nutrients are absorbed by intestinal epithelial cells via beneficial metabolic components. This process helps maintain intestinal microecology, preserves the integrity of the intestinal mucosal barrier, and prevents pathogens and toxins from entering the system.

Additionally, the Cluster of Differentiation 14 (CD14), an important innate immune receptor predominantly located on the surface of myeloid cells such as monocytes, macrophages, and neutrophils, collaborates with the T-regulatory system to maintain immune homeostasis. This balance involves regulatory T cells (T-Reg) and key inflammatory factors (TNF-α, IL-6, IL-1β, MPO) remaining in equilibrium [54]. Conversely, in patients with UC, an imbalance in intestinal microbiota—triggered by genetic or environmental factors—results in metabolic disorders and damage to the intestinal barrier. This imbalance also disrupts immune regulation, leading to an overproduction of pro-inflammatory factors (TNF-α, IL-6, IL-1β, MPO) and a corresponding decrease in the anti-inflammatory factor IL-10. This creates a sustained inflammatory response, contributing to the development of UC. Therefore, the mechanism by which probiotics alleviate UC primarily involves regulating the intestinal microecological balance, repairing the intestinal epithelial cell barrier, and reducing the release of immune factors [55].

#### 3.2.1. Regulating Intestinal Microecological Balance

The human intestine represents a highly complex microbial ecosystem, distinguished by its diversity and dynamics. Each individual’s intestinal microbiota is uniquely composed and influenced by factors such as genetics, diet, age, and geographic region [56,57]. While the microbiota of healthy individuals typically remains stable, significant alterations can occur due to long-term dietary changes, antibiotic use, or disease states. Various bacterial species, encompassing both beneficial and pathogenic bacteria, have evolved competitive mechanisms—referred to as antagonism—over extended periods. These mechanisms include competition for nutrients, adhesion sites, and the production of antibacterial substances. Maintaining this dynamic balance is essential for intestinal health [58]. An imbalance in this antagonism has been associated with UC, leading to a reduction in beneficial bacteria and an increase in pathogenic bacteria within the gut [30].

Recent studies indicate that co-administration of tetrapeptide from maize (TPM) with probiotics enhances the diversity of intestinal microorganisms in UC models, reduces the Firmicutes/Bacteroidetes (F/B) ratio, and increases the abundance of beneficial bacteria such as *Muribaculaceae*, *Alistipes*, *Ligilactobacillus*, and *Lactobacillus* [59]. Furthermore, biofilm cells of *Lactobacillus plantarum* LR-1 have been shown to enhance the abundance of *Eubacterium hallii*, *Salinimicrobium*, *Kocuria*, and *Candidatus Bacilloplasma* in UC models [60]. A combination of oral probiotics, including *Lactobacillus casei*, *Lactobacillus plantarum* P-8, and *Bifidobacterium animalis* subsp. *Lactis V9*, can prevent a decline in microbial diversity and richness by promoting beneficial bacteria such as *Eubacterium ramulus*, *Pediococcus pentosaceus*, *Bacteroides fragilis*, and *Weissella cibaria* in patients, compared to mesalazine, a conventional treatment for UC [61]. These findings suggest that probiotics can mitigate UC by fostering the growth of beneficial bacteria, restoring the balance of the intestinal microbiota, and reestablishing microecological homeostasis.

#### 3.2.2. Repair Intestinal Epithelial Cells and Intestinal Barrier Function

In patients with UC, chronic intestinal inflammation results in damage and apoptosis of intestinal epithelial cells. This damage leads to the downregulation of tight junction (TJ) protein expression, structural impairment, thinning of the mucus layer, and dysbiosis within the intestinal microbiota. The increase in pathogenic bacteria and the decrease in beneficial bacterial metabolites compromise barrier function, ultimately resulting in the disruption of intestinal mechanical barriers and a marked increase in intestinal permeability. This phenomenon, commonly referred to as intestinal leakage, triggers systemic inflammation and exacerbates local intestinal inflammation, creating a vicious cycle associated with the pathogenesis, severity, and recurrence of UC.

Therefore, preserving the integrity of intestinal epithelial cells and repairing intestinal barrier function is a pivotal therapeutic strategy for managing UC.

An increasing body of evidence indicates that specific probiotic strains can contribute to the repair of the intestinal barrier through various mechanisms. For example, *Lacticaseibacillus paracasei CCFM1222* has been shown to upregulate the expression of tight junction proteins ZO-1, occludin, and claudin-1 in mice, reversing dextran sodium sulfate (DSS)-induced mucosal and barrier dysfunction [62].

Similarly, *Lactiplantibacillus plantarum SC-5* has demonstrated the ability to reduce mucosal damage in DSS-induced colitis models and enhance the expression of occludin and claudin-1 [14]. The mechanisms by which probiotics enhance the TJ barrier are diverse. For instance, *Lactobacillus rhamnosus GR10* can prevent the dissociation of TJ proteins and E-cadherin by downregulating key proteins in the MAPK/MLCK/MLC pathway, thereby decreasing intestinal permeability [63]. Another study indicated that *Lactiplantibacillus plantarum NWAFU-BIO-BS29* increased the expression of mucins (MUC2, MUC3) and TJ proteins (occludin and claudin-1) in a mouse model, highlighting its significant capacity to strengthen the intestinal barrier [64]. Engineering strategies have also been employed to enhance the efficacy of probiotics. For example, engineered probiotics with a multifunctional coating derived from *Lactobacillus rhamnosus GG (LGG)* can reverse the decrease in the expression of ZO-1 and occludin and significantly reduce goblet cell damage compared to 5-aminosalicylic acid (5-ASA), thereby demonstrating a protective effect on the integrity of the colonic epithelium [65].

In addition to Lactobacillus, other probiotic strains have exhibited similar benefits. *Bacillus paralicheniformis HMPM220325* effectively mitigated the reduction in TJ proteins such as ZO-1, occludin, and claudin-1 at both the mRNA and protein levels. It has been shown to significantly restore colon length and protect goblet cells and the mucus layer [66]. Furthermore, in vitro studies have demonstrated that the supernatant of *Saccharomyces boulardii CNCM I-745* enhances the delivery of E-cadherin to the cell surface by redirecting endocytic E-cadherin. This process involves Rab11A-dependent recycling endosomes, which not only restore the attachment junction of intestinal cells but also reinforce the overall intestinal barrier function [67].

These findings suggest that probiotics can mitigate UC by elevating the levels of tight junction proteins and restoring the mucus layer, thereby reducing intestinal permeability and enhancing the intestinal barrier.

#### 3.2.3. Regulating Immune Response and Inflammatory Signaling Pathways

The immune response and inflammation play a critical role in the pathogenesis of UC. Typically, the intestinal immune system maintains a delicate balance between tolerating symbiotic bacteria and defending against pathogens. However, in patients with UC, this balance is disrupted due to a combination of genetic predisposition and environmental influences. As a result, the regulation of immune responses and inflammatory signaling pathways has become a fundamental aspect of UC management.

Recent studies utilizing murine models have demonstrated that the probiotic *Lactic acid bacteria LB-9* can significantly reduce colitis-induced apoptosis in intestinal epithelial cells (IECs), a process mediated by NF-κB and closely associated with the inflammatory factor TNF-α [68]. Similarly, *Lacticaseibacillus rhamnosus 1.0320* and its progeny exhibited notable remission effects in dextran sulfate sodium (DSS)-induced colitis models. This strain can inhibit key proteins in the TLR4/MAPK/NF-κB pathway (TLR4, MyD88, JNK, p38, and p65) and reduce the production of pro-inflammatory cytokines (TNF-α, IL-6, IL-1β) [69]. In addition to NF-κB, other inflammatory pathways are also modulated. *Leuconostoc mesenteroides DRC 1506* has been shown to decrease the levels of pro-inflammatory cytokines (TNF-α, cyclooxygenase-2 (COX-2), and inducible nitric oxide synthase (iNOS)) in colon tissue while significantly increasing the level of the anti-inflammatory cytokine IL-10, thereby mitigating intestinal tissue inflammation [70].

Another critical mechanism involves the regulation of immune cell differentiation. Ursodeoxycholic acid (UDCA), a metabolite of *Lactobacillus acidophilus* (LA), can activate the RapGap/PI3K-AKT/NF-κB pathway, promoting the differentiation of regulatory T (Treg) cells and inhibiting the polarization of pro-inflammatory M1 macrophages. This process decreases the secretion of pro-inflammatory factors such as IL-6 and TNF-α, reduces intestinal inflammation, and improves colonic pathological damage in UC mice [71]. The anti-inflammatory effects of probiotics may also be strain-specific and involve distinct metabolites. Studies have shown that *Bifidobacterium FL-276.1* and *Bifidobacterium FL-228.1* significantly alleviated DSS-induced colitis. The aryl hydrocarbon receptor (AHR) and nuclear factor erythroid 2-related factor 2 (NRF2) pathways were activated in the colon of mice, while the NLR family pyrin domain containing 3 (NLRP3) was downregulated throughout the intervention. Indole-lactic acid (ILA) emerged as a potential effector molecule that can upregulate ZO-1, Claudin-4, and Occludin through the AHR/NRF2/NLRP3 pathway to enhance epithelial barrier function, while simultaneously inhibiting the increase in TNF-α, IL-6, and IL-1β [72].

Through these mechanisms, probiotics not only reduce systemic inflammation but also offer indirect benefits for the emotional health of UC patients [73]. Chronic inflammation is a significant link between inflammatory bowel disease (IBD) and “inflammatory depression” (the comorbidity of anxiety and depression) [73]. Pro-inflammatory cytokines such as TNF-α, IL-6, and IL-1β can cross the blood–brain barrier, impacting neurological function and inducing emotional and behavioral changes. By inhibiting these inflammatory factors, probiotics may alleviate local intestinal inflammation and mitigate inflammation-driven neuropsychiatric symptoms, representing a core mechanism by which probiotics contribute to mental health in this context [73].

Furthermore, research has demonstrated that probiotics possess antioxidative stress properties, effectively scavenging reactive oxygen species (ROS) and mitigating oxidative stress damage to intestinal tissues. For instance, mice treated with *Limosilactobacillus fermentum E7* exhibited significantly reduced myeloperoxidase (MPO) activity and malondialdehyde (MDA) levels in the colon compared to mice treated with DSS, while activities of superoxide dismutase (SOD) and glutathione peroxidase (GSH-Px) were markedly elevated [74]. Additionally, a study involving *Lactobacillus casei* ATCC 393 indicated that its synthetic Biogenic Se nanoparticles (SeNPs) can alleviate ROS-mediated mitochondrial dysfunction via the Nrf2 signaling pathway, thereby protecting the intestinal epithelial barrier from oxidative damage [75].

In conclusion, current studies indicate that probiotics may alleviate UC through multiple mechanisms, including the regulation of intestinal microbiota, barrier repair, immune modulation, and antioxidation. However, most mechanistic studies remain confined to animal models, with insufficient clinical translation evidence. For example, while several studies have documented the expression of repair tight junction proteins in animal models using specific strains (such as *L. rhamnosus* GR-10 and *B. paralicheniformis* HMPM220325), their effects in humans and the consistency of specific signaling pathways remain unclear. Furthermore, significant variations in strain dosage and intervention duration across different studies complicate comparative analyses and limit the clinical applicability of mechanistic research findings.

## 4. The Application of Probiotics in the Alleviation of UC

The following sections summarize findings from several clinical and preclinical studies investigating the effects of probiotic interventions in UC. It is important to note that the evidence is derived from a limited number of studies, and results should be interpreted with caution pending further validation in larger cohorts.

### 4.1. Application of Saccharomyces boulardii in the Alleviation of UC in a Mouse Model

In this study, female C57BL/6 mice with UC were selected as the experimental subjects [76]. The mice were administered *Saccharomyces boulardii* via gavage for three weeks prior to being challenged with dextran sulfate sodium (DSS) to induce UC. Various physiological indices were monitored throughout the experiment. The results indicated that the *Saccharomyces boulardii* treatment effectively alleviated UC symptoms, including colon shortening, intestinal mucosal injury, and apoptosis of colon tissue cells. Further analysis revealed that *Saccharomyces boulardii* significantly reduced the levels of pro-inflammatory factors in serum and maintained intestinal microbial stability by increasing the abundance of *Porphyromonadaceae* in the mice [77]. Additionally, fructooligosaccharides (FOS), a commonly used prebiotic, selectively promote the growth of beneficial probiotics such as *Bifidobacterium* and *Lactobacillus* in the intestine while reducing the production of pro-inflammatory cytokines, thereby regulating intestinal immunity [78]. A recent study demonstrated that the combination of FOS and *Saccharomyces boulardii* produced superior anti-inflammatory effects in a mouse colitis model. This combination not only significantly reduced the disease activity index (DAI) and effectively inhibited colitis but also notably increased levels of butyric acid and isobutyric acid. Furthermore, the combination therapy significantly enhanced the abundance of beneficial bacteria, including *lactic acid bacteria* and *bifidobacteria*, effectively regulating the composition of the intestinal microbiome [76]. This research provides a solid scientific foundation for the prevention and alleviation of UC.

### 4.2. Application of Mixed Probiotics in UC Patients

This study involved a randomized controlled trial with 130 patients diagnosed with UC [79]. The participants were divided into two groups: 65 patients in the experimental group received a treatment regimen consisting of mesalazine, somatostatin, and a bifid triple viable capsule, while 65 patients in the control group were treated with only mesalazine and somatostatin. The bifid triple viable capsule used in the experimental group is a compound probiotic formulation that primarily contains three active probiotics: *Bifidobacterium longum*, *Lactobacillus acidophilus*, and *Enterococcus faecalis*.

The total effective rate was 92.31% in the experimental group compared to 76.92% in the control group. Regarding inflammatory markers, the experimental group exhibited lower levels of IL-6 (90.01 ± 8.02 ng/L vs. 101.27 ± 7.27 ng/L) and TNF-α (61.87 ± 6.38 ng/L vs. 68.29 ± 7.11 ng/L). Immune profiling revealed higher proportions of CD 4+ T cells (46.37 ± 4.21% vs. 40.02 ± 3.21%) and a higher CD4+/CD8+ ratio (1.79 ± 0.14 vs. 1.41 ± 0.09) in the experimental group. An indicator of intestinal barrier function, the D-lactic acid level, was also lower in the experimental group (4.01 ± 0.08 mmol/L vs. 4.61 ± 0.02 mmol/L). The incidence of adverse reactions was 4.62% in the experimental group and 7.69% in the control group. This study provides robust evidence for the use of probiotics as an adjunctive treatment for UC, underscoring its significant clinical relevance [79]. However, the independent contribution of the probiotic combination and its synergistic effects with conventional drugs require further investigation.

### 4.3. Application of Dietary Probiotics in UC Patients

In a study involving 93 UC patients, participants were randomized into a probiotics combination group (UC-P, *n* = 44) and a control group (UC-NP, *n* = 49) [80]. The UC-P group received a supplement containing five probiotics (*Bifidobacterium infantis*, *Bifidobacterium animalis*, *Lactobacillus bulgaricus*, *Lactobacillus helveticus*, and *Enterococcus faecalis*, each at ≥5 billion CFU), along with L-glutamine, biotin, and dietary guidance for 12 weeks.

After the intervention, the UC-P group demonstrated increases in muscle mass (MM) and skeletal muscle index (SMI), as well as a decrease in the extracellular water/total body water ratio (ECW/TBW). The Short Inflammatory Bowel Disease Questionnaire (SIBDQ) score improved from 45.21 to 60.47. The rate of abnormality in the inflammatory marker C-reactive protein (CRP) decreased from 50% to 9.1% [80].

This study not only confirmed the positive effects of the probiotic combination on intestinal symptoms in UC patients but also highlighted its significant role in regulating systemic inflammatory responses. Furthermore, the combination demonstrated beneficial effects on nutritional metabolism and body composition. These findings provide new insights into the use of probiotics for the comprehensive management of inflammatory bowel disease. They suggest that a specific strain combination may serve as an adjunctive management strategy to alleviate extraintestinal symptoms, such as muscle loss and metabolic disorders, in UC patients. The mechanisms of action and clinical application potential of this strategy warrant further investigation [80].

### 4.4. Application of Fecal Microbiota Transplantation in UC Patients

Fecal microbiota transplantation (FMT) is a therapeutic intervention designed to restore the balance of intestinal microbiota by transferring fecal microorganisms from healthy donors. This method introduces a diverse array of probiotics into the recipient’s body. In this study, ten patients with moderate to severe active UC underwent six sessions of FMT.

Clinical symptoms improved, as indicated by a significant decrease in the Truelove-Witts index (*p* < 0.05). Levels of inflammatory markers, including C-reactive protein (CRP) (*p* = 0.0004) and fecal calprotectin (*p* = 0.002), were reduced following FMT. Metagenomic analysis revealed alterations in the patients’ intestinal microbiota, characterized by an increased abundance of *Lactobacillus*, *Prevotellaceae*, and *Firmicutes*, alongside a decrease in *Staphylococcus* and *Bacillus* [81]. These findings suggest that multi-course FMT can effectively regulate intestinal microbiota in UC patients, alleviate inflammation, and is safe for clinical use. This approach presents a promising new therapeutic option for UC with broad application potential [81].

Despite the encouraging results from the aforementioned clinical studies regarding the use of probiotics in managing UC, several significant limitations persist: (1) The sample sizes are generally small (for instance, only ten cases in the FMT study); (2) The follow-up periods are often short (typically ≤12 weeks); (3) There is a lack of standardized strain combinations and dosages. For example, Li et al. (2021) demonstrated that triple probiotic capsules significantly improved inflammatory markers but did not clarify the independent contributions of each strain, nor did they establish a probiotic-only treatment group, complicating the assessment of its synergistic effects with conventional therapies [79]. Furthermore, most studies failed to perform stratified analyses based on the baseline characteristics of patients’ intestinal microbiota, overlooking the influence of individual differences on treatment efficacy.

### 4.5. Summary and Critical Appraisal of Evidence

The studies reviewed, while promising, exhibit considerable variability in design, quality, and scale, complicating the development of definitive clinical recommendations. To provide a comparative overview, key characteristics of selected studies are summarized in Table 2. The table illustrates that while several randomized controlled trials (RCTs) report positive outcomes with probiotic adjunct therapy [79,80], common limitations include small sample sizes, short study durations, and the frequent use of multi-component interventions, which hinder the isolation of the specific probiotic effects. The most robust evidence typically arises from meta-analyses, although these often highlight significant heterogeneity among trials [15,16]. Preclinical animal studies, while essential for elucidating mechanisms (as detailed in Section 3.2), cannot directly predict efficacy in humans. This appraisal emphasizes the need for larger, longer-duration, and more rigorously designed RCTs utilizing standardized probiotic preparations to advance the field toward reliable, evidence-based applications.

## 5. Prospect

The primary management strategies for UC include aminosalicylates, glucocorticoids, immunosuppressants, and biologics. However, these approaches present significant limitations in clinical practice. As illustrated in Table 3, aminosalicylates such as sulfasalazine, mesalazine, and olsalazine can assist in managing symptoms in patients with mild to moderate UC. Nevertheless, their ability to penetrate the inflamed mucosa is limited, reducing their effectiveness in severe cases [5,82,83].

Commonly utilized glucocorticoids include prednisone, prednisolone, and budesonide. While these medications can rapidly mitigate inflammation, their immunosuppressive effects can lead to adverse outcomes. Prolonged use may result in osteoporosis and an increased risk of fractures [84]. Additionally, complications such as diabetes and fatty liver, stemming from disturbances in glucose and lipid metabolism, can significantly impair patients’ quality of life [85,86,87]. Immunosuppressants, including azathioprine, mercaptopurine, methotrexate, and cyclosporine A, typically exhibit a delayed onset of action, often requiring 2–3 months to demonstrate efficacy. During this period, patients remain vulnerable to severe complications, such as intestinal perforation and bleeding, due to inadequate disease control. Furthermore, the risks of hepatorenal toxicity and bone marrow suppression associated with these treatments may lead to irreversible organ damage and serious hematological disorders [88,89].

Biological agents such as infliximab, adalimumab, and vedolizumab have transformed targeted treatment options; however, their prohibitive costs have deterred many patients. A single course of treatment can amount to tens of thousands of US dollars, imposing an economic burden on families that exceeds their financial capacity [8]. Additionally, approximately 30% of patients may develop drug resistance within 6 to 12 months of treatment, with some needing to discontinue therapy altogether due to allergic reactions [9,10]. Moreover, these conventional therapies often provide only symptom relief rather than a definitive cure for UC. The condition is prone to recurrence, necessitating long-term or even lifelong treatment, which imposes a substantial economic burden on both patients’ families and society at large [90].

Probiotics, as an emerging and rapidly advancing field, have witnessed significant progress in scientific research, industrial application, and public awareness in recent years. Their role has expanded beyond the traditional regulation of intestinal function to encompass a broader range of health domains, offering new avenues for the supportive management of UC. Probiotics effectively inhibit harmful bacteria by regulating intestinal microbiota balance and restoring the dominance of beneficial bacteria. Additionally, they activate intestinal immune cells and enhance mucosal barrier function, thereby improving overall intestinal defense.

In comparison to traditional medications, probiotics are characterized by high natural safety, lack of liver and kidney toxicity, and minimal risk of drug resistance, making them particularly suitable for vulnerable populations such as children and the elderly. Importantly, probiotics can continually improve intestinal microecology, potentially reducing recurrence rates of UC and shifting management strategies towards a more holistic approach that addresses underlying dysbiosis. This offers a more economical, safe, and sustainable supportive option for patients with UC.

While the long-term use of conventional medicine can adversely affect other bodily functions, its role during the acute phase of UC remains essential. In the early or remission stages of UC, it is advisable to establish healthy eating habits to support intestinal health, in conjunction with the use of probiotics to prevent disease progression. Following the acute phase of colitis, when the intestinal mucosal barrier, microbiota balance, immune function, and digestive capabilities are often still recovering, probiotics can provide comprehensive support.

Probiotics facilitate the repair of damaged intestinal mucosa. The short-chain fatty acids (SCFAs) they produce supply energy to intestinal mucosal cells and promote epithelial cell proliferation. Additionally, they competitively inhibit harmful bacteria from adhering to the mucosa and mitigate toxin-induced stimulation. Collectively, these actions enhance the integrity of the intestinal barrier, significantly reducing the risk of inflammation recurrence. To address the common imbalance of intestinal microbiota following the acute phase, probiotics can introduce beneficial exogenous bacteria, enhancing their population while suppressing the growth of harmful bacteria. Furthermore, probiotics restore microbial community diversity, improve intestinal metabolic function, and decrease endotoxin production, thereby effectively reducing systemic inflammatory load.

In terms of immune regulation, probiotics stimulate intestinal immune cells to secrete anti-inflammatory factors and inhibit the release of pro-inflammatory factors. They also promote mucus and immunoglobulin secretion, balance immune cell activity, alleviate chronic inflammation, and enhance mucosal immune defenses. Probiotics can relieve symptoms such as diarrhea or constipation by regulating intestinal peristalsis and breaking down undigested carbohydrates, which reduces gas production and abdominal distension. Additionally, they synthesize various vitamins, promote mineral absorption, and address malnutrition resulting from poor absorption. For patients with chronic colitis, long-term probiotic supplementation can mitigate antibiotic-related side effects and help maintain disease remission stability. Studies indicate that such interventions may also reduce the risk of serious complications, including intestinal mucosal carcinogenesis.

Although probiotics have demonstrated significant potential in the management of UC, this review identifies critical challenges that impede their standardized clinical application. Based on the current state of research and clinical needs, future probiotics research should concentrate on five key areas:

1. Treatment Standardization: It is imperative to develop evidence-based guidelines for precision medicine through systematic dose–effect studies and head-to-head comparative trials of different strains, such as *Lactobacillus* and *Bifidobacterium*, as well as specific combinations, such as *Bifidobacterium* triple viable capsules. Clarifying the optimal treatment parameters for various strains will provide a standardized solution for clinical practice.
2. Evidence Level Improvement: Promotion of large-scale, long-term (≥12 months) multi-center clinical trials is essential. These studies should aim to assess the long-term efficacy and safety of probiotics in maintaining clinical remission and reducing recurrence rates, thereby addressing significant gaps in the current evidence base.
3. Precision Medicine: The integration of multi-omics techniques (metagenomics, metabolomics, and host genetics) is needed to analyze the biological underpinnings of individual differences in treatment response. By constructing predictive models based on baseline intestinal microbiota and genetic characteristics, it will be possible to achieve individualized and accurate matching of probiotic regimens.
4. Synergistic Treatment Strategies: A systematic exploration of the synergistic mechanisms between probiotics and traditional first-line medications (such as mesalazine and biological agents) is necessary. This research should clarify optimal combination regimens and timing for different disease stages (acute and remission) to provide a practical treatment pathway for clinical application.
5. Management of Mental Health Comorbidity: Future research should focus on the impact of probiotics on emotional disorders in UC patients. It is advisable to use emotional assessment scores (e.g., HADS, PHQ-9) as secondary endpoints in clinical trials. The efficacy of specific strains, such as *Bifidobacterium longum CCFM1077* and *Lacticaseibacillus rhamnosus JB-1*, in alleviating anxiety and depression symptoms in UC patients should be systematically evaluated. By combining microbiome analysis, metabolomics, and neuroimaging techniques, the specific molecular and neural pathways through which probiotics regulate the gut–brain axis and improve mental health comorbidities can be elucidated. Ultimately, a personalized microecological treatment plan that integrates both physical and mental health will be developed for UC patients.

By systematically advancing these key areas, we anticipate overcoming the heterogeneity challenges in the current clinical application of probiotics and establishing their role as reliable, effective, and personalized components in the comprehensive management of ulcerative colitis.

## Figures and Tables

**Table 1:** The regulating effect of some probiotics on the intestinal tract.

Probiotics Genus	Strain	Physiological Function	Action Mechanism	Ref.
*Lactobacillus*	*Lactobacillus acidophilus*	Improve gastric disease	The secretion of lactic acid lowers intestinal pH, exerting a direct bactericidal effect.	[35,36]
*Lacticaseibacillus*	*Lacticaseibacillus rhamnosus*	Prevent intestinal diseases such as constipation	Regulate the expression of tight junction proteins in intestinal epithelial cells, enhance intestinal barrier function, and improve the balance of intestinal microbiota.	[37,38]
	*Lacticaseibacillus casei*	Reduce joint swelling and pain, and improve joint function	Activate intestinal immune cells, balance inflammatory factors	[39,40]
*Bifidobacterium*	*Bifidobacterium infantis*	Reduce liver inflammation, treat alcoholic liver injury	Regulate intestinal microbiota and enhance intestinal barrier function	[41,42]
	*Bifidobacterium longum*	Relieve emotional stress	Promote the proliferation of intestinal mucosal cells, enhance intestinal barrier function, regulate the secretion of intestinal neurotransmitters, and improve intestinal dysfunction.	[43,44]
*Bacillus*	*Bacillus subtilis*	Relieve abdominal distension, indigestion.	Secrete a variety of enzymes, such as protease, amylase, etc., to help break down food	[45,46]
*Saccharomyces*	*Saccharomyces boulardii*	Relieve diarrhea, constipation	Regulate intestinal microbiota, improve microbial ecology, and inhibit intestinal peristalsis by regulating intestinal microbiota.	[47,48]

**Table 2:** Summary of selected studies on probiotics in UC.

Study Type	Model/Population	Sample Size	Intervention (Probiotic Strain/Product)	Key Findings	Notes on Design/Evidence Level
Animal Study	DSS-induced colitis mice	Not specified	*Saccharomyces boulardii*	Alleviated symptoms, reduced pro-inflammatory factors, increased Porphyromonadaceae [75].	Preclinical proof-of-concept. Demonstrates mechanism but direct human applicability is limited.
Animal Study	DSS-induced colitis mice	Not specified	FOS + *Saccharomyces boulardii*	Superior anti-inflammatory effect vs. monotherapy, increased beneficial SCFAs and bacteria [76].	Suggests potential synergy with prebiotics.
Randomized Controlled Trial (RCT)	UC patients	130	Mesalazine + Somatostatin + Bifid Triple Viable Capsule (*B. longum*, *L. acidophilus*, *E. faecalis*) vs. control (no probiotics)	Higher clinical efficacy (92.31% vs. 76.92%), lower IL-6, TNF-α, D-lactate; better immune profile in probiotic group [79].	Moderate evidence. Suggests adjunctive benefit. Limitations: Probiotic not tested alone; specific strain contributions unclear.
Randomized Controlled Trial (RCT)	UC patients	93	Multi-strain probiotic (*B. infantis*, *B. animalis*, *L. bulgaricus*, *L. helveticus*, *E. faecalis*) + nutrients vs. control	Improved SIBDQ score, reduced CRP abnormality, positive effects on muscle mass and body composition [80].	Moderate evidence. Highlights systemic benefits. Limitations: Intervention includes nutrients (L-glutamine, biotin), making probiotic effect isolation difficult.
Clinical Study (Uncontrolled)	Moderate–severe active UC patients	10	Fecal Microbiota Transplantation (FMT)	Improved clinical index, reduced CRP and calprotectin, modulated microbiota (↑ Lactobacillus, ↑ Firmicutes) [81].	Low evidence/Preliminary. Promising but very small sample, no control group. Highlights feasibility and broad microbial modulation.
Meta-Analysis	Pooled analysis of multiple RCTs in UC patients	Varies (across studies)	Various probiotic strains and combinations	Probiotic supplementation may increase remission rates and is generally safe, but effects vary by strain and formulation [15,16].	High-level evidence synthesis. Strength varies with included study quality. Often reveals significant heterogeneity, underscoring need for standardized protocols.

Note: ↑ indicates an increase in content. This table summarizes a selection of studies discussed in the review for comparative purposes. It is not an exhaustive systematic review.

**Table 3:** Potential problems of different chemical drugs in the treatment of UC.

Drug Type.	Representative Drug	Defect	Ref.
Aminosalicylate preparations	Sulfasalazine, mesalazine, olsalazine	Limited efficacy in severe disease; may delay necessary intervention.	[5,82,83]
glucocorticoid	Prednisone, prednisolone, budesonide	Osteoporosis, increased infection risk, glucose and lipid abnormalities with long-term use.	[84,85,86,87]
immunosuppressant	Azathioprine, mercaptopurine, methotrexate and cyclosporine A	Slow onset; risk of hepatorenal toxicity and bone marrow suppression.	[88,89]
biologics	Infliximab, adalimumab, vedolizumab	High cost; potential for drug resistance or allergic reactions.	[8,9,10]

## Data Availability

Not applicable.
